# Service user involvement in the education of allied healthcare professionals in Ireland: a mixed-methods exploration

**DOI:** 10.1186/s12909-026-08575-3

**Published:** 2026-01-14

**Authors:** Sarah Dillon, Pauline Boland, Anne Griffin, Erin Adam, Elizabeth Helen Cooper, Paula Dowling, Nimra Parveen Ahmed, Aoife Lily Gallagher

**Affiliations:** 1 Health Research Institute (HRI), University of Limerick, Limerick, V94 T9PX Ireland; 2 School of Allied Health, Faculty of Education and Health Sciences, University of Limerick, Limerick, V94 T9PX Ireland

**Keywords:** Public engagement, Healthcare education, Service user

## Abstract

**Background:**

Service user involvement (SUI) in healthcare education is reported to foster more patient-centred practitioners. However, there is limited guidance as to how authentic SUI can be embedded within educational practice. This study aimed to describe current practices and explore how SUI may be better embedded in the delivery of healthcare education.

**Methods:**

A convergent mixed-methods study was undertaken in a higher-level healthcare education department in Ireland. Data were collected via an online survey completed by educators (*n* = 27) and semi-structured interviews with service users (*n* = 6). Survey data were analysed using descriptive statistics and content analysis. Thematic analysis of interview transcriptions was undertaken.

**Results:**

Most educators indicated that SUI was limited to ‘one-off’ interactions or case-based learning and that there was ‘probably’ (75%) or ‘definitely’ (9%) not enough SUI. Both staff and service users reinforced the value of SUI in healthcare education, yet the potential of the role of the service users was not fully realised. Service users described the benefits of their involvement in humanising patients beyond the context of their condition/illness. Several challenges were highlighted, including limited resources, lack of empowerment and insufficient knowledge of the scope of SUI.

**Conclusions:**

The need to educate all stakeholders about the potential for SUI beyond one-off contributions was highlighted. Additionally, engaged leadership is needed to facilitate the contextual integration of processes and procedures to embed the role of service users in higher education. Further investment is needed to advance SUI, which may require additional encouragement from funders, policy makers and regulators.

**Supplementary Information:**

The online version contains supplementary material available at 10.1186/s12909-026-08575-3.

## Background

Service user involvement (SUI) is increasingly recognised as a valuable element of healthcare education [1]. This mirrors a shift in the conceptualisation of the role of the service user in clinical practice and research, moving away from the role of passive recipient to partner in decision-making [1, 2]. SUI in healthcare education has, in part, been driven by developments in international policy [3, 4] and national guidelines [5, 6].

Aligned with the ethos of ‘nothing about us without us’ [7] SUI acknowledges the unique lived expertise of an individual’s personal, cultural and medical backgrounds in designing and delivering more relevant and effective educational experiences for healthcare practitioners in training [2]. The many benefits of SUI in medical education for students are well-documented. These include an increased holistic approach to healthcare delivery, better orientation to patient preferences, and enhanced empathic communication skills [8–10]. Service users similarly report benefits, which include increased levels of self-confidence, empowerment, and a sense of purpose associated with their involvement [11, 12]. Whilst SUI in health service design and research has received considerable attention [13, 14] this element of healthcare education has received relatively less [15].

Feedback from stakeholders, including service users, has become essential for most regulatory boards which accredit health professional training [16]. Authentic, sustainable SUI in the design and delivery of the curriculum is the goal [17]. This appears challenging to achieve in practice, where many learning encounters remain transactional in nature [2, 10, 18] and several barriers to such authentic and sustainable engagement exist [19–21]. To date, the nature and extent to which SUI has been implemented across healthcare education settings remain unclear [16], and there is limited guidance as to how more authentic and sustainable SUI practices could be embedded within this educational context.

### Study aims

This convergent mixed-method study aimed to describe current SUI practices within an allied healthcare department in an Irish University, and to explore how SUI may be better embedded in the delivery of healthcare education in this setting.

The following research objectives were addressed:

- To explore how educators describe SUI practices in the delivery of healthcare education within their programmes.
- To explore how service users describe their involvement in the delivery of healthcare education.
- To identify how authentic SUI could be embedded within healthcare programmes from the perspective of educators and service users.

## Methods

### Compliance with Ethical Standards

Ethical approval was granted from the Faculty of Education and Health Sciences Research Ethics Committee at the University of Limerick, Ireland (2023_05_12_EHS).

### Study Design

An exploration of SUI in healthcare education was undertaken using a convergent mixed-methods design [22]. Data were collected from two key stakeholder groups to gain a comprehensive understanding of the topic. A combined qualitative-quantitative survey was conducted, in addition to qualitative interviews, to serve complementary and expansionary purposes[22, 23]. The survey of educators allowed for quantification of the involvement of service users, with qualitative questions embedded to provide deeper insights. Semi-structured interviews with service users were completed to explore their experiences in relation to healthcare education in depth. Findings from both phases were integrated during interpretation.

### Research Team

The research team comprised eight female researchers. Of these, four were academics within the field of occupational therapy (PB), speech and language therapy (ALG), human nutrition and dietetics (AG) and physiotherapy (SD). The remaining four researchers were postgraduate students undertaking professional healthcare qualifications (EA, NA, EHC, PD).

### Setting and context

This study took place in the School of Allied Health, within the University of Limerick, one of seven publicly funded universities in the Republic of Ireland. The school offers a range of undergraduate and postgraduate healthcare professional qualification programmes, including occupational therapy, physiotherapy, speech and language therapy, and human nutrition and dietetics. All professional programmes are approved by the health regulatory body of Ireland (CORU).

### Sampling and recruitment

Purposive sampling and snowballing techniques were employed. For the survey, all academic and practice education staff within an Allied Health School were recruited, primarily via email, bolstered by word of mouth. For the recruitment of interviewees, educators acted as gatekeepers, inviting their current network of eligible service users.

### Data collection and analysis

#### Online survey

A custom web-based survey was created using Qualtrics software and circulated via email. Questions were mainly quantitative, with some opportunities to add additional free text (Appendix 1). They focused on current practices, drawing on key areas of SUI identified from the literature [16], as well as views on barriers and facilitators of authentic SUI [15]. Following the design period, the survey was piloted, drawing on the interdisciplinary experiences of the research team. Two people not involved in the core research team also piloted the survey. Feedback was sought on comprehension, coherence and length of the questionnaire, as well as testing the online functionality of the survey. Primary changes included adding more ‘other’ boxes where participants could input additional information and adjusting phrasing to maximise readability. Data collection for the survey occurred between August and September 2023. Survey responses were downloaded from Qualtrics to Microsoft Excel (Version 2401, Microsoft) and anonymised. Descriptive statistical analysis of quantitative data and content analysis [24] of free text data was undertaken.

#### Semi-structured interviews

Semi-structured interviews with service users were held online via Teams (Microsoft, 2024). A topic guide was developed based on a review of key literature on the topic (Appendix 2). All interviews were recorded, transcribed and uploaded to NVivo (Version 14, Lumivero). Braun and Clarke’s six steps were used to guide analysis [25, 26]. Three research meetings were held between ALG, NA, and EA to generate codes and to identify central organising themes. Each researcher familiarised themselves with the data by listening to the audio files whilst reading the transcript. Reflexive notes were recorded in NVIVO in relation to coding decisions, where thematic development progressed iteratively with reflections from the experienced and novice researchers enhancing reflexivity. Findings of the study were circulated to service users for review and comment. One service user requested that the importance of clear instructions around the logistics of involvement be highlighted. This was integrated into the results. No further changes were suggested.

#### Data analysis

Findings from both stakeholder groups were evaluated using Towle’s Spectrum of Engagement [2] (Table 1), which enables an understanding of the extent to which SUI practices involve power-sharing between educators and service users, an essential element of authentic SUI. The use of this framework also enhanced the transferability of our findings by providing levels of involvement against which activities and practices elsewhere can be compared. Types of SUI reported by educators in the survey or service users during interviews were mapped to corresponding levels of involvement.

**Table 1:** Towle’s spectrum of involvement [2]

Towle Level	Degree to which SU is actively involved in learning encounter
1	Paper-based or electronic case or scenario.
2	Standardised or volunteer patient in a clinical setting.
3	Patient shared their experience with students within a faculty-directed curriculum.
4	Patient-teacher(s) are involved in teaching or evaluating students.
5	Patient-teacher(s) as equal partners in student education, evaluation and curriculum development.
6	As (5) above plus, patient(s) involved at the institutional level.

## Results

### Survey

Twenty-seven educators completed the survey, from a potential pool of thirty-six, representing a 75% response rate, reflecting strong engagement from participants and enhancing the credibility and internal validity of the survey findings [27]. Questions were not compulsory, so not all questions were answered. As such, response rates vary by question. Educator demographics are provided in Table 2.

**Table 2:** Respondent characteristics of educator respondents (*n* = 27)

*Background Details*	*n (%)*
Programme	
Occupational therapy	8 (30%)
Physiotherapy	11 (41%)
Speech and language therapy	5 (19%)
Human nutrition and dietetics	6 (22%)
Intermediary studies	1 (4%)
Role in the school*	
Course director	8 (30%)
Module lead	15 (56%)
Discipline lead	2 (7%)
Practice educator	8 (30%)
Other	2 (7%)
Length of time in current role	
Less than two years	8 (30%)
Two to five years	11 (40%)
More than 5 years	8 (30%)

*Some respondents reported holding more than one role (e.g., course director and module lead) and indicated this in their survey responses. As a result, the role counts presented may exceed the total number of individual respondents

#### Classification of educator-reported SUI

**Fig. 1:**
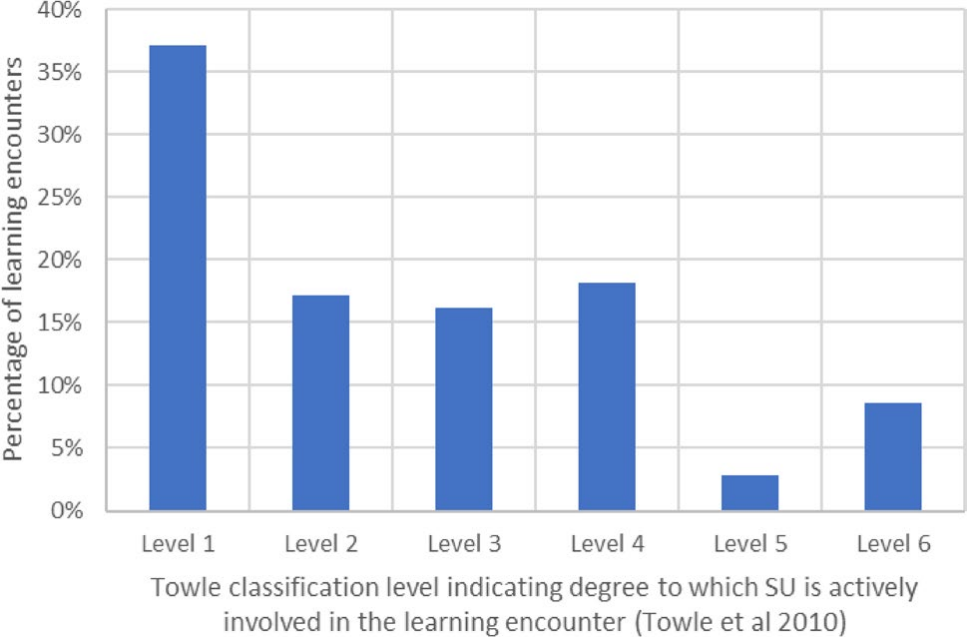
Service user (SU) involvement in educator-reported learning encounters based on Towle’s Spectrum of Involvement

Learning encounters were categorised and mapped to their corresponding level (Fig. 1). The majority of interactions were at the lower levels of Towle’s Spectrum; 88% were classified level 1 to 4, and of these, 37% were at level 1. Only 12% of encounters included curriculum development (level 5) or involvement at an institutional level (level 6). For most cases (31%), SUI took the form of direct encounters with students, such as guest lecturing. Indirect methods (26%), such as generic case studies or pre-recorded videos and presentations, were also commonly reported.

Eighty-four percent of educators indicated there was‘probably’ (75%) or ‘definitely’ (9%) not enough SUI within their modules. Eleven percent of educators, most of whom were involved in practice education, reported that SUI featured consistently across their modules. In relation to the service user role in curriculum decision-making, 25% of educators reported that service users had little say in what or how they contributed to the programme, and 13% of educators reported that service users had “a lot” of autonomy. Around half of the respondents described a process of shared decision-making between service users and educators. Educators were asked about collecting and disseminating feedback from both service users and students about SUI. Feedback about SUI was mainly sought in an ad-hoc and informal manner, with educators requesting student feedback on SUI sessions in 35% of cases, and service users being asked for their feedback in 74% of cases. Dissemination of feedback to colleagues was either informal or did not occur, with only 17% of educators presenting this during quality assurance feedback routes such as staff or course board meetings. 

#### Barriers to authentic and sustainable service user involvement from the educator perspective

The majority of educators (84%) described a lack of value placed on SUI in healthcare programmes at the institutional level. Due to a lack of institutional support, educators were concerned that engagement could be tokenistic and even harmful. Twenty two percent of educators reported that monetary support was offered to service users for their contribution, though the majority did not receive reimbursement. Educators stated that there were limited formalised processes in place to reimburse service users. This was viewed as unacceptable, as many service users incurred personal costs when contributing to the programmes. Furthermore, financial processes for service user reimbursement, where they existed, were described as time-consuming and unwieldy. Although many educators expressed an appetite for more sustained SUI in education, six educators viewed SUI as an adjunct to teaching and learning, rather than integral to curriculum content. Other practical university-level barriers included delays with release of timetables and related inflexibility. Transport was a challenge to arrange, especially where service users needed to travel a long distance.

#### Facilitators of authentic and sustainable SUI from the educator perspective

Educators recognised the value of stable, long-term relationships between service users and educators to ensure authentic and sustainable involvement. The idea of developing a service user panel was proposed as one means of supporting such relationships. Panel members could have a set term and formalised processes of recruitment to enhance sustainable relationships and increase the diversity of service users involved. Maintenance of communications, ideally with a single primary staff contact across the programmes, with a member of the panel, was suggested to streamline the processes of engagement. The establishment of the panel would ensure continuity of involvement across modules where there could be yearly changes change of lead educator. 

The provision of appropriate training for staff and service users was endorsed in the open-text responses by 15 educators to support authentic engagement. An understanding of university structures was identified as an important element of service user training to support full participation in curriculum decision-making. Supportive administrative processes and systems would enable educators to embed SUI in their practice more easily. Examples of supportive administrative processes included arrangements regarding transport and parking, as well as remuneration processes. Time to engage with service users outside of the classroom was identified as important, but would need to be balanced with other priority roles and responsibilities.

### Semi-structured interviews

#### Service user characteristics

Six participants were recruited for the interviews, service users (*n*=5) and a family member of a service user (*n*=1). The exact number of service users targeted for recruitment is unknown, as invitations were distributed via educators’ networks; however, it is estimated that more than 20 service users contribute to programmes across the school. Although service users were reportedly involved across all disciplines, no service user involved in the human nutrition and dietetics programme participated. The gender, lived experience, background, and professional group service users contributed to are outlined in Table 3. Interviews were an average of 32 minutes in duration.

**Table 3:** Description of service user characteristics

ID Code	Background	Gender, lived experience	Programme Involvement
P1	Professional athlete *Service user*	Male, personal experience of Cerebral palsy	Physiotherapy
P2	Accountant *Service user*	Female, personal experience of Parkinson’s Disease	Physiotherapy
P3	Teacher *Mother of a Service User*	Female, personal experience as mother of child with a developmental delay	SLT & OT
P4	Salesperson *Service User*	Male, personal experience of stroke	SLT patient-led scheme
P5	*Service User*	Male, personal experience of stroke	SLT patient-led scheme
P6	Academic *Service User*	Female, personal experience of traumatic brain injury	OT

*Abbreviations OT  Occupational Therapy, SLT * Speech and Language Therapy

#### Level of involvement

Four of the six service users who were interviewed described their experiences at Level 3, where the service user experience with students sits within a faculty/regulator-directed curriculum (Table 1). Two service users reported involvement in the co-design and co-delivery of a communication partner scheme within the speech and language therapy professional qualification programme, more aligned with shared decision-making of Level 4.

Five themes were identified: (i) Including the service user experience can humanise disability for students (ii) SUI needs to be supported; (iii) SUI is challenging at many levels; (iv) SUI can be mutually beneficial, and (v) Service users want to give back, to raise awareness and to promote inclusion.

(i) Including the service user experience can humanise disability for students
  Service users believed that hearing personal stories helped students empathise with a patient, highlighting the real-life impacts of living with health conditions. Some service users communicated the importance of being listened to and being heard: 
  …because students can see me. And what I have to do to talk, what I have to do to walk. (P4)
  Service users felt that their involvement had the power to shift the student perspectives, where students may have had less exposure to hearing from people living with health conditions and that their contribution can ensure the student sees the patient as a whole person:
  I suppose it's [to] kind of see the person as a person and not just different parts of the body… (P2)
(ii) SUI needs to be supported
  The need for SUI to be supported was heavily referenced. Receiving both formal and informal training before interacting with students was helpful:
  So, I got training before the summer break on how to give feedback to SLT students. And when the students got back last month, I started putting that training into practice. (P5)
  Service users appreciated clear guidance ahead of their sessions regarding how best to deliver their talks and orientation to the physical layout of the campus. Outlining the topic focus for student learning, as well as descriptions of the class size and clear instructions around location, parking and accessibility were each deemed to be important, with such information reducing initial fear or uncertainty. A lack of support in being oriented to buildings and rooms was described as problematic:
  Because sometimes, like in people with conditions like myself, stress can be can kind of bring on a bit of anxiety and stuff like that. Trying to navigate getting to a lecture hall of a university you don’t know can be a bit of challenge when you’re living with something that affects your movement. As a five-minute walk could take ten minutes for some. (P2)
  Communication support and the need to explore preferred communication methods for service users from a speech and language therapist was important:
  I think it would be very important if, if you had someone with speech difficulties delivering a module or teaching a lecture, a guest lecture or whatever to be offered the opportunity to engage with speech and language therapy and that would enhance the confidence of the person to engage. (P6)
  Participants highlighted the importance of feedback between service users, students and educators:
  Feedback from the students that what you've said, has this really put them to sleep or it's of some interest or benefit, so it's (good) to get a bit of feedback as well. (P2)
(iii) SUI is challenging at many levels
  Theme (iii) encapsulates the challenges that many service users face when engaging in educating healthcare professionals. Personal factors for service users, such as anxiety and stress, influenced their involvement. Different health conditions are associated with a variety of symptoms that increase such challenges. For example, one service user said:
  Fatigue would be a challenge for me and, you see, it fluctuates. And so I, I recorded the… a snippet of what I was going to say the night before. (P6)
  Many service users were involved in single presentation-based encounters. Just one participant saw a role for service users in developing curricula. However, beyond these one-off interactions as guest lecturers, service users struggled to identify how else they could have contributed meaningfully to the programmes:
  I suppose devising you know the curricula and things is probably beyond most users, you know, and kind of specialty in itself. (P3)
  A reason behind this reaction could be that most service users did not consider themselves to have the required skillsets to participate in higher levels of involvement. When asked about further involvement beyond one off sessions, one service user said:
  I wouldn’t have the knowledge or the criteria to do that, obviously. (P1)
  The fact that service users bring a variety of opinions and experiences was discussed as potentially challenging. For example, one service user described how access to services differed across Ireland, and that each service user may have differing experiences:
  I’m only, you know, one parent and all the parents will have their experiences and it, you know, can vary a lot between the different regions as well. (P3)
(iv) SUI can be mutually beneficial
  Theme (iv) captures the benefits of SUI, not only for the service users themselves, but also for the students. The appreciation of the varying interactions and social aspects of the participants’ involvement was mentioned as *“rewarding”* (P4). The connections made throughout were also seen as mutually beneficial, as it opened doors for not only the service users to learn, but also for the future healthcare professionals to increase their knowledge. One service user reported that their involvement offered “on the ground” knowledge that could enhance programmes and future health service delivery, which would benefit both students and service users:
  The more you involve people with insider knowledge… that can tell you the truth with what was happening on the ground, you know, and that is very beneficial because you can improve the service delivery and also make them [service users] feel involved where they're more motivated to give you that in-depth insight into how you can improve it and inform policy as well in the area. (P6)
(v) Service users want to give back, to raise awareness and to promote inclusion.
  Theme (v) expresses the service users' desire to give back to the communities, healthcare professionals and students, to raise awareness, and to facilitate inclusivity. Service users expressed desires to advocate for themselves and for others with their health condition, as well as promote opportunities available for their peers:
  …giving back to students and the teachers, because I never [would] be there without the students and the teachers and speech therapists. (P4)
  For many, SUI was rooted in helping others understand their experiences and the insider knowledge that comes with being a service user. Service users cited that engaging with students provided them with a platform to express their views, thoughts, and concerns regarding their condition/diagnosis. By acknowledging the*“…value in educating others (P3*)”, they were able to promote inclusivity.
  When you have lived experience of using a service and it can change, the outlook or perspective of the whole program, and it might make it more inclusive for certain people. (P6)

## Discussion

This mixed-methods study explored the current practices and perspectives of educators and service users in embedding SUI in allied health education at an Irish university. The views of educators were collected from an online survey, and service user perspectives were gathered from semi-structured interviews.

### State of play- current SUI practices

This study found limited SUI reported, despite openness to this approach from educators and well-documented benefits, consistent with previous research [1]. Most educators indicated that the current extent of SUI was insufficient, with contributions generally aligned to the lower levels of Towle’s Spectrum of Involvement [2]. This is likely due to the barriers, outlined by both groups, such as logistical challenges for reimbursement and practical support, as reported elsewhere [21, 28]. Similarly, limited SUI is common in other institutions, contributing minimally to student assessment and curriculum development [10, 15, 18, 29]. Lower-level SUI in isolation risks a perception of inauthentic and tokenistic partnership [10], a fear echoed by educators in our survey. Educators noted that SUI is not suitable for every module, emphasising the need for context-appropriate integration into healthcare education at the discretion of individual educators.Similarly, most service users indicated that they felt that they were not appropriately positioned to contribute beyond recounting their experience, a role which can be limited [18].

### Buying into the vision: empowering service users to contribute

SUI in education can be framed as empowerment, grounded in the philosophy of ‘nothing about us without us’ [7], and the principle that service users have the prerogative to educate about their experiences and embodied knowledge [30]. Thus, service users are regarded as equal partners, fostering a culture of co-collaboration. However, a universal approach to achieve this is yet to be established. Adequate training and support have been proposed to contribute to the implementation and legitimacy of SUI [31]. In our study, both service users and educators acknowledged the value of clear guidance and training to support SUI in programmes. However, the lack of awareness of the service user’s potential role at Towle’s Level 4 or greater (curricular/institutional level) signals a lack of empowerment beyond lower levels of involvement.

Previous research has similarly indicated that limited knowledge of existing programme delivery and content hindered service users’ capacity to identify where their input could be incorporated into the curriculum [32]. Moreover, educators in this study were protective of their ownership of curricular development, mirroring previous research [33]. Thus, it may be necessary to develop formal roles with defined scopes and tailored support for service users, as institutional language and knowledge may be required for effective contribution [31]. This is best directed by service users themselves, rather than “imposed” upon them [34], with sufficient flexibility to facilitate the service users’ distinctive expertise [35]. Although models have been suggested to achieve this [32], further application and evaluation in practice are needed.

### Embedding service user involvement: an implementation science lens

Our findings suggest that authentic, collaborative and sustainable SUI has not yet been embedded in healthcare education. Similarly, a report by the UK’s Health and Care Professions Council identified gaps in SUI implementation in higher education, noting that some service users were weakly involved through supplementary measures, rather than part of a sustained strategy [36]. Drawing on concepts from implementation science, embedding SUI requires contextual integration, which is essential in normalising new practices [37]. In our study, much of the engagement was led by individual practitioners, rather than an organised system and formalised process. This practice likely contributes to the inconsistencies in SUI within and between institutions.

Institutional support and a culture of openness are key to embedding SUI in healthcare education [35]. Applying an implementation science lens considers factors influencing the successful adoption of innovations such as SUI. In this context, compatibility relates to how well innovations integrate with existing workflows and systems, and it has been demonstrated to have an important influence on implementation outcomes [38, 39]. Embedding sustained SUI will require flexible and responsive processes regarding how, where, and when such engagement happens. For example, some service users in our study valued more informal and diverse methods of communication when engaging with educators, in contrast with the formal administrative processes and procedures that can characterise the university setting. Balancing the need to formalise service user engagement in decision-making with the flexibility of how it occurs will be challenging and will require additional resources. Clearly, issues of compatibility and fit require commitment beyond individual service users and local educator champions. Studies across several educational contexts indicate that engaged leadership can help overcome many of these challenges [40, 41]. Therefore, the commitment and engagement of senior decision-makers are essential for the successful integration of SUI.

### Driving SUI: policy and incentives

While research funding typically provides clear guidance and expectation of SUI in healthcare research as mandatory, this is not replicated in healthcare education. Several practical suggestions for guiding future practice emerged from this study. Amongst service users, comprehensive guidance, thoughtful logistical planning that considers health-related limitations, and constructive feedback to service users were recommended. Amongst educators, the development of a panel of service users to consult on the development of healthcare programmes, administrative support, reimbursement, and training were identified as necessary. Underpinning enhanced SUI is investment of resources, such as funding and dedicated planning time. Insights from health research, where advocacy, policies and funding incentives have mandated and rewarded public and patient involvement [14]. While momentum is emerging for SUI, further promotion, incentives, and formal mandates may be necessary. to strengthen education institutional commitments to embedding SUI as standard.

### Strengths and limitations

This work offers a nuanced overview of SUI at one public university in the Republic of Ireland from both educator and service user perspectives. A strength of this study is its interprofessional perspective on SUI. Likewise, interprofessional learning opportunities should be considered when further embedding service users, maximising the potential for SUI to enhance collaborative practice, whilst simultaneously lessening demands on service users to deliver sessions to individual groups. This research also has several limitations. Firstly, although open-ended questions and free text boxes allowed for the sharing of opinions by educators, in-depth nuances and unanticipated insights could be further identified using interview or focus group methods. Secondly, we aimed to include service users with a range of experiences of healthcare access, but it was not possible to capture the insights of all types of service users. Future research could benefit from a dedicated service user contact database. Thirdly, although students contributed to the design and analysis of this study, further research is needed to explicitly examine their perspectives on SUI, a focus now underway. Finally, we acknowledge a missed opportunity to involve service users as co-researchers.

## Future directions and conclusions

Both service users and educators recognised the beneficial impact that SUI has on health education and service users themselves. However, several barriers to embedding sustainable and authentic engagement were identified, suggesting that the implementation of such initiatives is challenging in the context of higher education institutions. The leadership of organisations is crucial in developing a feasible, context-specific implementation strategy that enables service users to engage fully as partners in shared decision-making. There may be a need for stipulated/mandated requirements for educational institutions, such as policy directives and incentives, to encourage the standard embedding of SUI in healthcare education. Our findings can inform future development of consensus-based best practice guidelines. In the spirit of collaboration and partnership, many stakeholder voices should be included to build towards a future of improved healthcare education. 

## Supplementary Information

Supplementary Material 1.

Supplementary Material 2.

## Data Availability

The datasets generated and/or analysed during the current study are not publicly available in line with the ethics application and consent form associated with the study.

## Abbreviations

SUI: Service user involvement
