# Supplementary material for: Service user involvement in the education of allied healthcare professionals in Ireland: a mixed-methods exploration
Source: BMC Med Educ. 2026 Jan 14;26:234. doi: 10.1186/s12909-026-08575-3 (PMC12888578; doi:10.1186/s12909-026-08575-3)
Supplement: Supplementary file 1 — Supplementary Material 1. [file 12909_2026_8575_MOESM1_ESM.docx]

**Appendix 1**

Survey for academic staff

Q 0 Do you agree to proceed with this survey?

- Yes, I will proceed (1)
- No, I do not wish to proceed (2)

Q1 Please indicate your role at the University (select as many options as apply)

- Course director
- Module lead
- Discipline lead
- Practice educator
- Other (please specify)

Q2 Please indicate area of specialization / interest (select as many options as apply)

- Occupational therapy
- Physiotherapy
- Speech and language therapy
- Human nutrition and dietetics
- Intermediary studies
- Advanced healthcare practice

Q3 What is the duration of your current role at the School of Allied Health? (please select one option)

- Less than two years
- Two to five years
- More than five years

Q4 Is there service user involvement in every module in your programme, and if not please explain why you believe this is the case?

*Free text response.*

Q4a Of the modules that you led in academic year 2022/2023, please detail below i) the module name, ii) the module code, iii) the number of service users involved, and iv) which person is involved (i.e. service user themselves, service user family member, service user support worker, or another stakeholder-please be specific). Please note: all text boxes below must be filled to progress to the next question - enter N/A where appropriate.

|  | i) Module name (1) | ii) Module code (2) | iii) Number of service users (3) | iv) Persons involved (4) |
| --- | --- | --- | --- | --- |
| Module 1 (8) |  |  |  |  |
| Module 2 (9) |  |  |  |  |
| Module 3 (10) |  |  |  |  |
| Module 4 (12) |  |  |  |  |

Q5 How are service users involved in your programme? (select as many options as apply)

- Generic paper based / electronic case study, based on your professional / clinical experience
- Paper based / electronic case study, based on discussion with a service user, or prepared by a service user
- Lecture content contribution without face-to-face teaching (e.g., pre-recorded video or presentation)
- Scripted service user encounter (e.g., service user provides a history, or student practices an examination)
- Service user shares their experience within a faculty directed curriculum, planned by faculty member, but within level of comfort of the service user
- Service user freely shares their experience with little faculty direction
- Guest teaching (one off)
- Guest teaching (series)
- Co-teaching with academic staff
- Group workshops (e.g., 4-8 Service Users per class of approximately 30 students)
- Face-to-face interactions with students
- Virtual (live) interactions with students
- Providing informal feedback to students
- Assessment design
- Assessment administration, including evaluation (as a model / contributing to assessment feedback)
- Involvement across the curriculum as a whole (rather than just one module)
- At institutional level in the decision making for healthcare education / policy making
- Accreditation and/or CORU involvement
- Research partners within the academic curriculum (e.g. in FYPs)
- Contribution to research evidence within the academic curriculum (e.g., in FYPs)
- Other (please give details)
- No service user involvement

Q6 How have you, in your role at the School of Allied Health, identified the service users currently engaged in your programme? (select as many options as apply)

- By word of mouth through my family or friends
- By word of mouth through an additional role I hold / have held (e.g. current research role; previous clinical role)
- Via voluntary body / associated charity
- Service User approached the School of Allied Health
- Through my research links
- Link was initiated before I commenced this role
- There is no service user involvement
- Someone else is responsible for service user engagement
- Other (please specify)

Q7 In what ways are service users supported to teach, and / or reimbursed for this role at the SCHOOL OF ALLIED HEALTH? (select as many options as apply)

- Formal Education (e.g. enrolment in a course or module at University of Limerick - please specify)
- Informal Education (e.g. guidance on learning outcomes of lecture or module; public speaking tips) with university contact (e.g. module lead; practice educator)
- Informal Education with another member of the School of Allied Health teaching staff
- Financial reimbursement for time and travel expenses through expense claim
- Financial reimbursement for time and travel expenses in vouchers / gift cards
- Refreshments provided as part of their visit
- "Meet and greet" with academic contact or School of Allied Health teaching staff
- Having a support person attend (paid or unpaid)
- Inclusion in / connection with a service user involvement support group at the University of Limerick (if yes, please identify the group)
- They are not reimbursed
- They are not supported
- Other (please specify)

Q8  In your experience, how do service users engaged in your programme prefer to be referred to? (select as many options as apply)

- Service users
- Participants
- Stakeholders
- Experts by experience
- Lived experience contributors
- Collaborators
- Co-tutors
- It depends on their situation (please give details)
- I have never formally or informally asked this
- Other (please explain)

Q9 To what extent do service users direct what, and how, they teach on the programme? (please select one option and explain)

- A little (freetext response)
- A lot (freetext response)
- It is a negotiation between learning outcomes and their confidence (freetext response)
- Other (freetext response)

Q10 Do you believe that there is sufficient service user involvement on your programme? (please select one option)

- Definitely not
- Probably not
- Unsure
- Probably yes
- Definitely yes

Q10a In what ways would you like to see more service user involvement in your programme?

*Free text response.*

Q11 What supports do you believe would be required to expand/consolidate the role of service users in your programme? (e.g. supports from the University, CORU, service users, students etc.)

*Free text response.*

Q12 What challenges do you foresee or have you witnessed in service user involvement in the design, delivery or assessment of your programme?

*Free text response.*

Q13 In what way is feedback collected from STUDENTS detailing their experience of interacting with a service user? (select as many as apply)

- UL online questionnaire without a deadline (e.g. guest lecture satisfaction surveys / module satisfaction surveys)
- UL online questionnaire with a specific deadline
- Hard copy UL questionnaire at the end of the session
- Both hard copy and online options are offered
- Feedback is not consistently requested
- Online MCQ link
- Other (please specify)

Q13a Approximately, what percentage of STUDENTS reply to requests for feedback on service user involvement?

|  | 0 | 10 | 20 | 30 | 40 | 50 | 60 | 70 | 80 | 90 | 100 |
| --- | --- | --- | --- | --- | --- | --- | --- | --- | --- | --- | --- |

| % of Students who provide written feedback () | 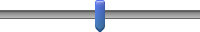 |
| --- | --- |

Q14 In what way is feedback collected from SERVICE USERS detailing their experience of interacting with both students and School of Allied Health staff? (select as many as apply)

- Written questionnaire
- MCQ
- Feedback is given verbally and recorded by the lecturer
- Feedback is not consistently requested
- Feedback if collected, is done in an ad-hoc / informal manner e.g. during a coffee and chat with the key contact
- Other (please specify)

Q14a Approximately what percentage of SERVICE USERS reply to requests for feedback on their experience of interacting with both students and School of Allied Health staff.

|  | 0 | 10 | 20 | 30 | 40 | 50 | 60 | 70 | 80 | 90 | 100 |
| --- | --- | --- | --- | --- | --- | --- | --- | --- | --- | --- | --- |

| % of Service Users who provide written feedback () | 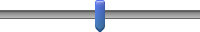 |
| --- | --- |

Q15 In what way do you record and share feedback of service user and student interactions in your programme within the School of Allied Health? (select as many as apply)

- I record and share informally in an ad-hoc manner
- I have not shared feedback
- I store written feedback / MCQ results and only share when requested
- I am not aware of an identified / agreed pathway for feedback sharing
- Other (please specify)

Q16 If you have any additional comments that you would like to share, please enter them here.

*Free text response.*

Q17 Will you act as a gatekeeper for phase two of this study, where service users are invited to participate in an online focus group to share their experiences of service user involvement with the School of Allied Health? Please select one option. 
(You are required to send invitations on behalf of the School of Allied Health to the service users you engage with in your programme.)

- Yes
- No
- I would like to discuss this further with a researcher
- I do not lead the co-ordination of service user involvement
