# Supplementary material for: Service user involvement in the education of allied healthcare professionals in Ireland: a mixed-methods exploration
Source: BMC Med Educ. 2026 Jan 14;26:234. doi: 10.1186/s12909-026-08575-3 (PMC12888578; doi:10.1186/s12909-026-08575-3)
Supplement: Supplementary file 2 — Supplementary Material 2. [file 12909_2026_8575_MOESM2_ESM.docx]

**Appendix 2**

Topic Guide- Interview

1. Can you tell us about how you first became involved with the School of Allied Health?
2. Can you tell us about how you contribute – how often, to what students and about what expertise?
3. What kind of initial information/support/training did you receive for your role in this school, if you can remember? How might this have been improved?
4. What do you enjoy about this role?
5. Is there anything that you find difficult or frustrating about this role?
6. What could the School of Allied Health do to better support you (if anything)?
7. What do you think of the current level/amount of service user involvement in the curriculum of school of allied health?
8. How else would you like to see service users like yourself involved in the education of future healthcare professionals?
9. Is there anything else you would like to add?
